# Meteorin-Like Protein (Metrnl) in Obesity, during Weight Loss and in Adipocyte Differentiation

**DOI:** 10.3390/jcm10194338

**Published:** 2021-09-23

**Authors:** Andreas Schmid, Thomas Karrasch, Andreas Schäffler

**Affiliations:** Department of Internal Medicine III, Giessen University Hospital, 35392 Giessen, Germany; thomas.karrasch@innere.med.uni-giessen.de (T.K.); andreas.schaeffler@innere.med.uni-giessen.de (A.S.)

**Keywords:** meteorin-like protein, Metrnl, adipokine, obesity, bariatric surgery, low calorie diet, adipose tissue, adipocyte, fatty acids

## Abstract

Meteorin-like protein (Metrnl) is an adipo-myokine with pleiotropic effects in adipose tissue (AT). Its systemic regulation in obesity and under weight loss is unclear. Circulating Metrnl concentrations were analyzed by ELISA in severely obese patients undergoing bariatric surgery (BS) or low calorie diet (LCD). Metrnl mRNA expression was analyzed in human and murine tissues and cell lines by quantitative real-time PCR. About 312 morbidly obese individuals underwent BS (*n* = 181; BMI 53.4 + 6.8 kg/m^2^) or LCD (*n* = 131; BMI 43.5 + 6.7 kg/m^2^). Serum samples were obtained at baseline and 3, 6, and 12 months after intervention. AT specimen from subcutaneous and visceral adipose tissue were resected during BS. Serum Metrnl levels were lower in type 2 diabetic patients and negatively correlated with HbA1c. In BS and LCD patients, Metrnl concentrations significantly increased after 3 months and returned to baseline levels after 12 months. There was no gender-specific effect. Metrnl mRNA expression did not differ between visceral and subcutaneous AT in *n* = 130 patients. In contrast, Metrnl gene expression in mice was highest in intra-abdominal AT followed by subcutaneous, peri-renal, and brown AT. In the murine 3T3-L1 cell line, Metrnl expression was high in pre-adipocytes and mature adipocytes with a transient downregulation during adipocyte differentiation. Metrnl expression remained unaffected upon treatment with glucose, insulin, fatty acids, bile acids, and incretins. Polyunsaturated omega-3 and omega-6 fatty acids downregulated Metrnl expression. Systemic Metrnl is transiently upregulated during massive weight loss and gene expression in adipocytes is differentially regulated.

## 1. Introduction

The adipose tissue represents an endocrine and immunological organ with pleiotropic functions exerting an important role in whole body metabolism with a highly significant clinical impact [1,2]. Its systemic functions are mediated by a number of various secretory peptides and proteins (adipokines) that are involved in diverse physiological processes, particularly in metabolism, inflammation, and immunity [1,3,4,5,6]. In metabolic disorders such as obesity, immuno-modulatory adipokines have an essential role in the regulation of metabolically induced inflammation (“metaflammation”; “adipose inflammation”) [7,8,9].

**Figure 1:**
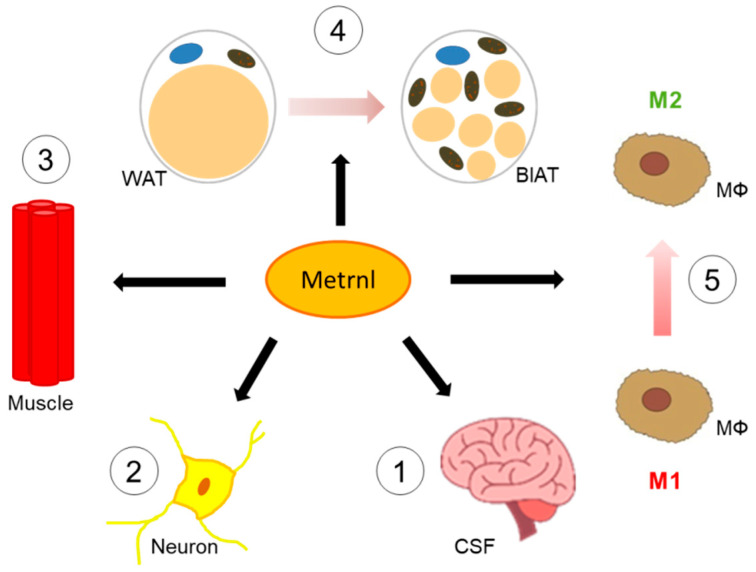
Biological functions of Metrnl in different tissues and cell-types. Metrnl is secreted by myocytes or adipocytes, appears in the cerebrospinal fluid (CSF) of humans (**1**) and crosses the blood-brain-barrier (BBB) [12]. Concentrations of Metrnl in CSF are similar to those in serum and increase with deterioration of BBB. In the central nervous system, Metrnl acts as a neuroprotective and neurotrophic factor (**2**) [11]. Regeneration of injured muscle is improved by Metrnl (**3**) [15]. Metrnl promotes “browning” processes in white adipose tissue (WAT), i.e., transformation of “classical” adipocytes toward a brown-like adipose tissue (BlAT) with thermogenic activity (**4**) [13]. This is supported by alternative activation of macrophages induced by Metrnl (**5**) [13]. Metrnl, Meteorin-like protein; MΦ, macrophage.

Meteorin-like protein (Metrnl; subfatin) represents a secretory protein with a molecular weight of ~30 kDa and a neurotrophic factor homologous to meteorin [10] and has a role in neuroblast migration and neuroprotection [11]. Of note, this protein is abundant in cerebrospinal fluid with its concentrations depending on blood-brain-barrier function [12]. In addition to various organs and tissues (digestive tract, skin, lung, brain), Metrnl is expressed with high levels in activated monocytes, skeletal muscle (post-exercise), and adipose tissue. Taken together, Metrnl has been characterized and regarded as an exercise-inducible myokine as well as an adipokine [13,14]. Regeneration of injured muscle is promoted by Metrnl via Stat3/IGF-1 signaling [15]. Figure 1 summarizes biological functions of Metrnl.

Most interestingly, post-exercise Metrnl expression in muscle is linked to PGC1-α expression promoting white adipose tissue browning. Metrnl is associated with alternative macrophage activation favoring anti-inflammatory and thermogenic processes in adipose compartments [13,16]. Thus, Metrnl can be considered an exercise- and cold-inducible adipo-myokine mediating beneficial effects by adipocyte-immune cell and by muscle-fat crosstalk [13].

Recent studies suggested beneficial effects of Metrnl in conditions such as chronic colitis [17], cholesterol and triglyceride homeostasis [18], chronic obstructive pulmonary disease [19], coronary artery disease [20], and insulin resistance [21]. However, currently published data on systemic Metrnl concentrations in obesity or type 2 diabetes mellitus (T2D) are somewhat controversial. Recently, a meta-analysis evaluating data from nine cohort studies could not identify a general association of circulating Metrnl levels with T2D [22], due to a high number of potential confounding variables. Of note, a recent study reported a protective role of Metrnl in diabetic mice without obesity [23]. While Metrnl plasma concentrations were found to be elevated in human individuals suffering from T2D and obesity [24], Pellitero et al. reported lower circulating levels in a small cohort of 25 obese patients. Another study demonstrated a subsequent increase of Metrnl levels following laparoscopic sleeve gastrectomy [25]. A recent study of diet-induced obesity in rats revealed increased Metrnl protein concentrations in muscle and white adipose tissue after sleeve gastrectomy, whereas circulating concentrations were found to be decreased [26]. Taken together, studies in obese humans are scarce and small-sized or even lacking regarding circulating Metrnl concentrations during long-term follow up of obese patients after bariatric surgery or under diet. Thus, the essential interest of the present study was to investigate these issues in a large study cohort comprising 312 morbidly obese individuals undergoing either bariatric surgery (*n* = 181 patients) or a multidisciplinary, life-style intervention program including low calorie diet (LCD; *n* = 131).

In particular, we focused on the quantification of:

- Circulating Metrnl concentrations (before intervention) with respect to correlate them with anthropometric and biochemical parameters;
- Metrnl gene expression in subcutaneous and visceral adipose tissue compartments of morbidly obese patients undergoing bariatric surgery;
- Circulating Metrnl concentrations longitudinally over 12 months following bariatric surgery or start of LCD;
- Metrnl gene expression in murine adipose tissues and in the murine 3T3-L1 cell line upon treatment with metabolites such as glucose, insulin, fatty acids, bile acids, and incretins.

## 2. Materials and Methods

### 2.1. Adipocyte Cell Culture and Stimulation Experiments

**Figure 2:**
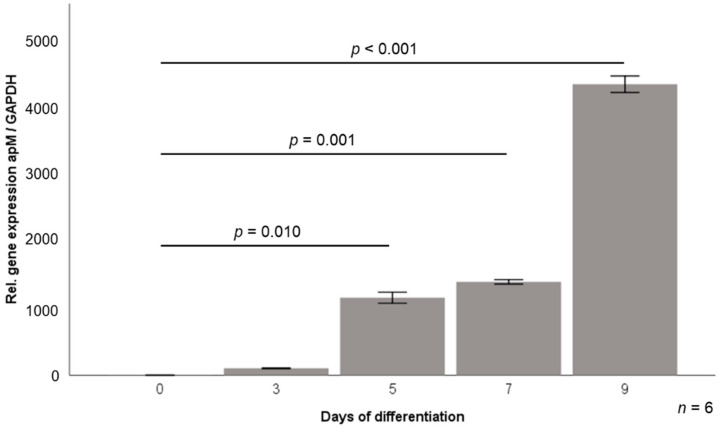
Relative gene expression levels of adiponectin during 3T3-L1 adipocyte differentiation. apM, adiponectin; GAPDH, glyceraldehyde-3-phosphate dehydrogenase.

3T3-L1 pre-adipocytes [27] were cultured and differentiated into mature adipocytes as described previously [28]. Briefly, cells were cultured at 37 °C and 5% CO_2_ in Dulbecco’s modified Eagle medium (Biochrom AG, Berlin, Germany) supplemented with 10% newborn calf serum (Sigma-Aldrich, Deisenhofen, Germany) and 1% penicillin/streptomycin (Aidenbach, Germany) and were differentiated into adipocytes in DMEM/F12/glutamate medium (Lonza, Basel, Switzerland) supplemented with 20 µM 3-isobutyl-methyl-xanthine (Serva, Heidelberg, Germany), 1 µM corticosterone, 100 nM insulin, 200 µM ascorbate, 2 µg/mL transferrin, 5% fetal calf serum (FCS, Sigma-Aldrich, Deisenhofen, Germany), 1 µM biotin, 17 µM pantothenic acid, 1% penicillin/streptomycin (all from Sigma Aldrich, Deisenhofen Germany), and 300 µg/mL Pedersen-fetuin (MP Biomedicals, Illkirch, France) [29,30]. A differentiation protocol reported in the literature [27,31,32,33,34] was used with slight modifications, with light-microscopy control of adipocyte phenotype. In addition, induced gene expression of adipocyte markers such as adiponectin during the differentiation process was verified applying real-time PCR (Figure 2).

Mature adipocytes were incubated under serum-free conditions prior to stimulation experiments. FFA were purchased from Sigma-Aldrich (Deisenhofen, Germany) and dissolved in 10% BSA/EtOH in stock concentrations of 200 mM. Palmitic acid (PA; 100 µM), stearic acid (StA; 100 µM), myristic acid (MyA; 100 µM), lauric acid (LaA; 100 µM), oleic acid (OA; 10 µM), linoleic acid (LiA; 10 µM), palmitoleic acid (PoA; 10 µM), arachidonic acid (ArA; 10 µM), eicosapentaenoic acid (EPA; 10 µM), and docosahexaenoic acid (DHA; 10 µM) were used for overnight (18 h) stimulation experiments (*n* = 6 wells each). All stimulating doses had been determined by previous experiments in adipocyte culture with respect to dose effects and cell viability [35]. In addition, cells were incubated under low/high glucose (5.56 mM/25 mM) concentrations and under low/high (0.2 and 2.0 nM) insulin concentrations. Among bile acids, cholic acid (CA; stimulation dose: 100 µM), deoxycholic acid (DCA; 10 µM), and ursodeoxycholic acid (UDCA; 50 µM) were used for stimulation. Among incretin hormones, glucagon-like peptide-1 (GLP-1; 50, 100, 200 nM) and glucose-dependent insulinotropic polypeptide (GIP; 100 nM) were investigated and purchased from Sigma-Aldrich (Deisenhofen, Germany) and were applied in overnight (18 h) stimulation experiments in mature 3T3-L1 adipocytes. LDH (lactate dehydrogenase) concentration was measured in supernatants (Cytotoxicity Detection Kit, Roche, Mannheim, Germany) from all cell culture experiments in order to exclude any unexpected cytotoxic effects.

### 2.2. Preparation of mRNA and Real-Time PCR Analysis of Metrnl Gene Expression in Murine Cells and in Murine and Human Adipose Tissue

Subcutaneous and visceral adipose tissue specimens were obtained from patients during bariatric surgery. Intra-abdominal and subcutaneous adipose tissue compartments were resected from wild-type C57BL/6 mice (bred under standard conditions and chow diet; sacrificed for organ samples conformable to *§4 Abs. 3 Tierschutzgesetz*). A specific announcement was made at the local ethical committee (*Regierungspraesidium Giessen*: internal registration number: 544_M) that was approved subsequently. Small portions of fresh intra-abdominal and subcutaneous adipose tissue were digested with 0.225 U/mL of collagenase NB 6 (#17458, SERVA Electrophoresis; Heidelberg, Germany) and adipocytes were separated from stroma-vascular cells (SVC) via centrifugation. Total mRNA was isolated from frozen human and murine total adipose tissues, and from cultured 3T3-L1 adipocytes as described previously [28]. Briefly, tissues were homogenized in TRIzol^®^-Reagent (Life Technologies GmbH, Darmstadt, Germany) in combination with gentleMACS dissociator and M-tubes (Miltenyi Biotec GmbH, Bergisch Gladbach, Germany) for dissociation and RNA was isolated applying RNeasy^®^ Mini Kit (Qiagen, Hilden, Germany) including DNase digestion (RNase-Free DNase Set, Qiagen, Hilden, Germany). For gene expression analysis, reverse transcription of RNA (QuantiTect Reverse Transcription Kit from Qiagen, Hilden, Germany) was performed in order to generate corresponding cDNA for real-time PCR (RT-PCR) (iTaq Universal SYBR Green Supermix, CFX Connect RT-PCR system; Bio-Rad, Munich, Germany). Expression levels of the target gene Metrnl were normalized by _ΔΔ_CT method to the gene expression of glyceraldehyde-3-phosphate dehydrogenase (GAPDH) which had been applied before as a reliable house-keeping gene for white adipose tissue and adipocytes by our group and by others [28,36]. The following primers were used:

Human Metrnl: 5′-AGTGGATGTACCCAACAGGTG-3′/5′-TACCAGCAGTCTCAGTTCTCC-3′

Human GAPDH: 5′-GAGTCCACTGGCGTCTTCAC-3′/5′-CCAGGGGTGCTAAGCAGTT-3′.

Murine Metrnl: 5′-CTGGAGCAGGGAGGCTTATTT-3′/5′-GGACAACAAAGTCACTGGTACAG-3′

Murine GAPDH: 5′-TGTCCGTCGTGGATCTGAC-3′/5′-AGGGAGATGCTCAGTGTTGG-3′.

All oligonucleotides used were purchased from Metabion (Martinsried, Germany).

### 2.3. ROBS (Research in Obesity and Bariatric Surgery) Study Cohort

Serum samples and specimens from subcutaneous (abdominal) and visceral (intra-abdominal) adipose tissue were collected from the *ROBS* (*Research in Obesity and Bariatric Surgery*) study cohort. *ROBS* is an open-label, non-randomized, monocentric, prospective, and observational (explorative and confirmatory) study of patients routinely undergoing either bariatric surgery (gastric sleeve or Roux-en-Y gastric bypass) or a low calorie formula diet (LCD) in the tertiary care center at the University of Giessen, Germany. The detailed information about this study cohort can be drawn from a recent publication [37] and basic characteristic are summarized in Table 1. Briefly, patients were treated by a multidisciplinary team of physicians and professionals from Internal Medicine, Endocrinology/Diabetology, Metabolic/Visceral Surgery, Psychosomatic Medicine/Psychotherapy, Nutritional Science/Dietetics, and Sports Medicine at the Obesity Centre at the University of Giessen, Germany. The study was approved by the local ethical committee at the University of Giessen, Germany (file: *AZ 101/14*). All patients gave informed consent and were informed about the aim of the study. Data anonymization and privacy policy were accurately applied. Obese patients with a BMI ≥ 40 kg/m^2^ or with a BMI ≥ 35 kg/m^2^ and coexisting type 2 diabetes were consecutively admitted for bariatric surgery from January 2015 to April 2021. Exclusion criteria were: pregnancy, evidence of or suspicion on underlying endocrine diseases, untreated bulimia nervosa and binge eating behavior, use of illicit drugs, neoplasm, severe psychiatric disorders, psychosis, and psychopathologic instability.

### 2.4. Measurement of Serum Metrnl Levels

Metrnl serum concentrations were measured in duplicates by ELISA (DuoSet ELISA development systems, R&D Systems, Wiesbaden, Germany) and are expressed as means ± standard deviation. The assay detection range was 15.6–1000 pg/mL.

### 2.5. Statistical Analysis

For explorative data analysis, a statistical software package (SPSS 26.0) was used. Metrnl concentrations did not follow a Gaussian distribution. Non-parametric numerical parameters were analyzed by the *Mann–Whitney U*-test (for 2 unrelated samples), the *Kruskal–Wallis* test (>2 unrelated samples), the *Wilcoxon* test (for 2 related samples), or the *Friedman* test (>2 related samples). A *p*-value below 0.05 (two tailed) was considered as statistically significant. In the figures, means are displayed as bars with whiskers giving the standard error of the mean (1 × SEM). Box plots are indicating median, upper/lower quartiles, interquartile range, minimum/maximum values and outliers.

## 3. Results

### 3.1. Quantification of Baseline Metrnl Serum Levels in Patients Undergoing LCD or Bariatric Surgery

Circulating Metrnl concentrations in morbidly obese patients were quantified by ELISA prior to weight loss induced by either LCD (*n* = 131; 88 females, 43 males; BMI = 43.48 ± 6.74 kg/m^2^) or bariatric surgery (*n* = 181; 143 females, 38 males; BMI = 53.36 ± 6.84 kg/m^2^). Mean baseline Metrnl serum concentrations were 1117 ± 378 pg/mL (range: 392–3840 pg/mL) in the LCD cohort and 1143 ± 383 pg/mL (range: 91–3786 pg/mL) in patients undergoing bariatric surgery (Table 1).

**Figure 3:**
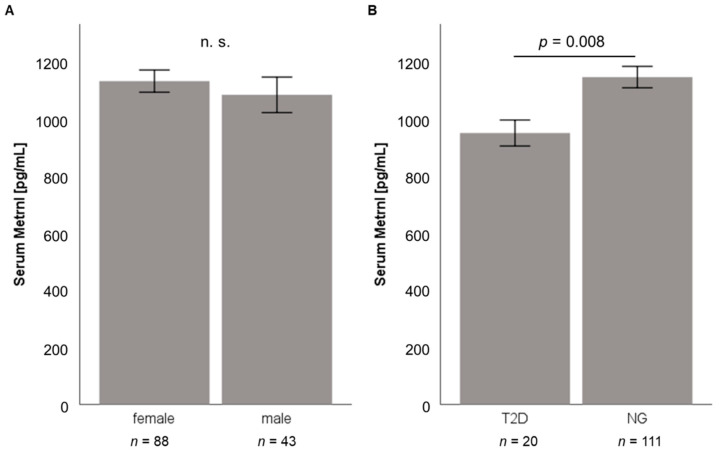
Subgroup analysis of baseline serum Metrnl concentrations in the LCD study cohort (*n* = 131). Serum Metrnl concentrations do not differ between male and female patients (**A**) and are significantly reduced in patients with type 2 diabetes mellitus (**B**). LCD, low calorie diet; Metrnl, Meteorin-like protein; NG, normal glucose tolerance (non-diabetic patients).

**Figure 4:**
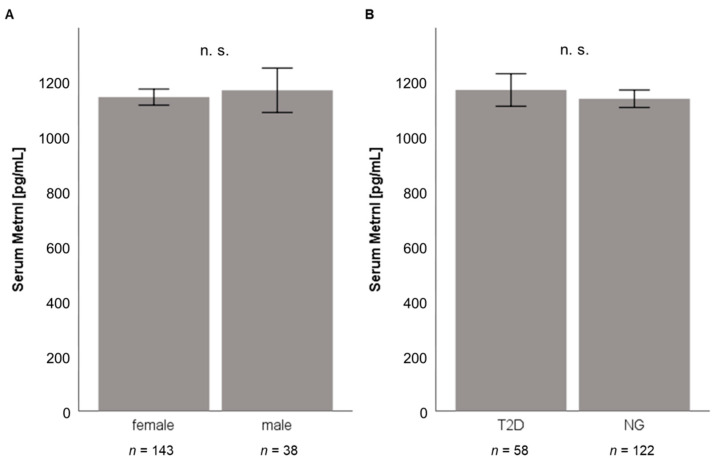
Subgroup analysis of baseline serum Metrnl concentrations in the bariatric study cohort (*n* = 181). There are no significant differences of serum Metrnl concentrations between male and female patients (**A**) and between T2D and non-diabetic patients (**B**). Metrnl, Meteorin-like protein; NG, normal glucose tolerance (non-diabetic patients); T2D, type 2 diabetes mellitus.

As illustrated in Figure 3A and Figure 4A, no significant gender-specific differences in Metrnl concentrations were observed in both cohorts, also neither in normoglycemic nor in T2D patients. Baseline Metrnl concentrations were not correlated with BMI or percentage body fat mass (Table 1). Within the LCD cohort, Metrnl levels were significantly decreased in individuals with type 2 diabetes mellitus (T2D) (Figure 3B) whereas this association could not be demonstrated in pre-bariatric patients (Figure 4B) which had a higher BMI than patients undergoing LCD.

Data analysis applying the *Spearman rho* test revealed significant correlations of circulating Metrnl concentrations with different biochemical parameters, classical adipokines, novel immune-regulatory adipokines, and growth factors (Table 2). Of note, a significant negative correlation between Metrnl and HbA_1c_ was found exclusively among LCD participants (rho = −0.269, *p* = 0.002) (Table 2). This finding fits very well with the lower Metrnl levels observed in T2D patients in this cohort (Figure 3B). Furthermore, there was a non-significant trend of a negative correlation between circulating Metrnl levels and HbA_1c_ for T2D patients among LCD participants (*n* = 20; rho= −0.425, *p* = 0.062) unlike normoglycemic LCD individuals (*n* = 111; rho= −0.151, *p* = 0.113). A more detailed subgroup analysis for the BS cohort stratifying for HbA_1c_ and BMI values revealed a slight yet significant, negative correlation of Metrnl with HbA_1c_ levels in patients with an HbA_1c_ < 6.5% but not for individuals with higher HbA_1c_ percentages. Regarding BMI subgroups, Metrnl and HbA_1c_ were negatively correlated exclusively in BS patients within a BMI interval ranging from 40.0 to 49.9 kg/m^2^ (rho = −0.267, *p* = 0.012).

In contrast to HbA_1c_ levels, triglycerides were negatively correlated with Metrnl only in bariatric patients (rho = −0.164, *p* = 0.037) whereas there was a positive correlation of HDL cholesterol and Metrnl levels in LCD (rho = +0.191, *p* = 0.029) as well as in bariatric patients (rho = +0.180, *p* = 0.022) (Table 2). Among classical adipokines, serum leptin (LCD: rho = +0.269, *p* = 0.002; BS: rho = +0.222, *p* = 0.003) and resistin (LCD: rho = +0.187, *p* = 0.032; BS: rho = +0.316, *p* < 0.001) were positively correlated with circulating Metrnl in both study cohorts. Subgroup analysis revealed that these correlations were significant for both males (leptin: rho = +0.226, *p* = 0.043; resistin: rho = +0.237, *p* = 0.033) and females (leptin: rho = +0.201, *p* = 0.002; resistin: rho = +0.263, *p* < 0.001) in the whole study cohort. Furthermore, a slightly positive correlation of Metrnl with the immune-regulatory adipokine progranulin was observed exclusively in LCD patients, whereas there were no significant correlations with C1q/TNF-related protein-3 (CTRP-3), cathelicidin antimicrobial peptide (CAMP) and fibroblast growth factors (FGF) 19 and 21 (Table 2) in both cohorts.

### 3.2. Metrnl Gene Expression Is Not Different between Visceral and Subcutaneous Adipose Tissue in Morbidly Obese Patients

**Figure 5:**
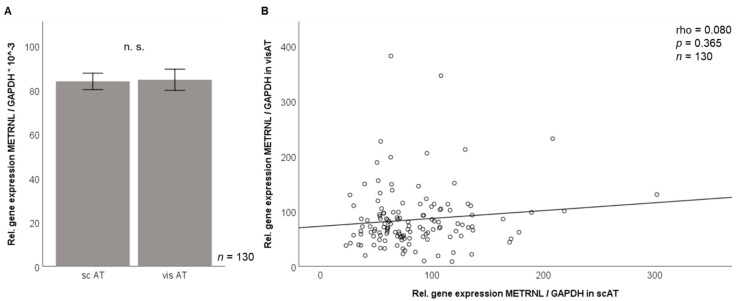
Metrnl gene expression in adipose tissue compartments (bariatric study cohort, *n* = 130). Metrnl mRNA expression levels in subcutaneous and visceral adipose tissue do not differ (**A**) and are not correlated with each other (**B**). GAPDH, glyceraldehyde-3-phosphate dehydrogenase; METRNL, Meteorin-like protein; sc AT, subcutaneous adipose tissue; vis AT, visceral adipose tissue.

Metrnl mRNA expression levels in human subcutaneous and visceral adipose tissue (*n* = 130) were analyzed by RT-PCR (_ΔΔ_CT method applying normalization to GAPDH expression). We did not detect significant differences in Metrnl gene expression levels of both fat compartments (Figure 5A) in this large array of human adipose tissue samples. Furthermore, visceral and subcutaneous adipose tissue Metrnl mRNA levels were not correlated (Figure 5B).

### 3.3. Correlation of Adipose Tissue Metrnl Gene Expression with Anthropometric and Biochemical Parameters

There were no significant differences in Metrnl mRNA levels between male and female patients in subcutaneous and visceral adipose compartments. Subcutaneous adipose tissue Metrnl gene expression was positively correlated with body weight (rho = 0.224, *p* = 0.010), BMI (rho = 0.182, *p* = 0.038), and homeostasis model of insulin resistance (HOMA-IR) (rho = 0.226, *p* = 0.027). Metrnl mRNA expression in visceral adipose tissue was positively correlated with waist-hip ratio (rho = 0.193, *p* = 0.042); data not shown.

Metrnl expression in visceral adipose tissue was positively correlated with progranulin serum levels (rho = 0.249, *p* = 0.009) and negatively correlated with circulating adiponectin (rho= −0.202, *p* = 0.022) and leptin concentrations (rho= −0.265, *p* = 0.002); data not shown. Of note, there was a negative correlation between subcutaneous adipose tissue CTRP-3 expression and the expression of Metrnl, both in subcutaneous (rho = −0.198, *p* = 0.026) and in visceral adipose tissue (rho = −0.205, *p* = 0.020); data not shown.

### 3.4. Serum Metrnl Levels Are Transiently Elevated during Early Stages of Weight Loss

Longitudinal serum samples from follow-up visits were available from 80 LCD participants (visits: 3, 6, and 12 months after beginning of dietary intervention (V3, V6, V12); mean weight loss after 12 months: 30.6 ± 15.2 kg) and from 82 bariatric patients (visits: 3–5 days post-surgery and 3, 6, and 12 months after bariatric surgery (V1, V3, V6, V12); mean weight loss after 12 months: 55.4 ± 17.0 kg).

**Figure 6:**
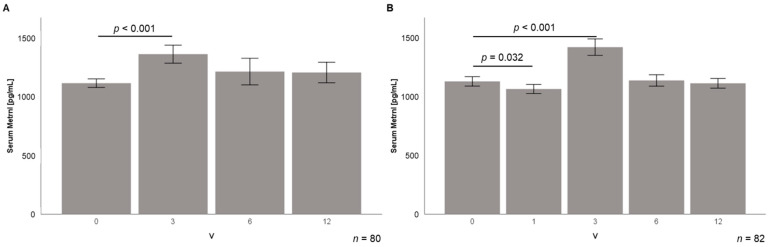
Transient upregulation of circulating Metrnl levels during longitudinal observation of weight loss in LCD (*n* = 80) and bariatric study cohort (*n* = 82). Metrnl serum concentrations are significantly elevated 3 months after start of LCD (**A**) or bariatric surgery (**B**). In the bariatric patients, there is an initial decline occurring directly upon surgery (3–5 days). LCD, low calorie diet; Metrnl, Meteorin-like protein.

Weight loss induced by LCD was associated with an initial elevation of circulating Metrnl quantities from 1112 ± 327 pg/mL to 1356 ± 689 pg/mL 3 months after the beginning of dietary intervention (*p* < 0.001) (Figure 6A). At the following visits (6 and 12 months), Metrnl concentrations returned to baseline levels (1206 ± 786 pg/mL after 12 months) (Figure 6A).

In the study cohort of bariatric patients, baseline serum Metrnl concentrations were 1126 ± 367 pg/mL and decreased immediately after surgery to 1057 ± 355 pg/mL (*p* = 0.023) within 3–5 days (Figure 6B). This initial decline of Metrnl levels was followed by a transient increase 3 months after surgery up to 1419 ± 646 pg/mL (*p* < 0.001), similarly to the LCD cohort (Figure 6B). Levels afterwards returned to baseline quantities at 6 to 12 months.

### 3.5. Correlation Analysis of Serum Metrnl during Weight Loss

Correlation analysis of circulating Metrnl concentrations with anthropometric and physiological parameters during stages of weight loss—3, 6, and 12 months after the beginning of weight reducing intervention (i.e., V3, V6, and V12)—was performed on the lines of regression analysis of baseline serum levels and patients characteristics as reported above (Table 2). Metabolic systemic parameters such as blood glucose and lipids were only assessed at baseline and V12 according to the study protocol. As is displayed in Table 3, no significant correlations of serum Metrnl with the assessed anthropometric and biochemical parameters were detected at study time-points V3 and V6 in both cohorts. Twelve months after the beginning of the diet program, there was a negative correlation of Metrnl with BMI in the LCD cohort (rho = −0.227, *p* = 0.043) (Table 3A). In bariatric surgery patients, Metrnl levels were positively correlated with blood glucose (rho = +0.232, *p* = 0.040) and with CRP levels (rho = +0.254, *p* = 0.023) (Table 3B).

### 3.6. Murine Tissue Expression of Metrnl mRNA

**Figure 7:**
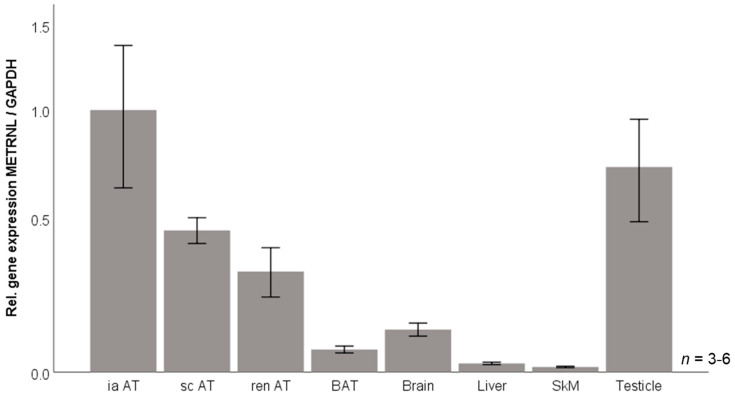
METRNL gene expression in murine tissues. BAT, brown adipose tissue; GAPDH, glyceraldehyde-3-phosphate dehydrogenase; ia AT, intra-abdominal adipose tissue; METRNL, Meteorin-like protein; ren AT, peri-renal adipose tissue; sc AT, subcutaneous adipose tissue; SkM, skeletal muscle.

Against the background of data for human circulating and adipose tissue expressed Metrnl in obesity, we aimed to investigate its regulation under defined experimental conditions in the established murine 3T3-L1 adipocyte model in vitro. Prior to this cell culture approach, basal Metrnl gene expression levels in vivo were assessed for different organs and tissues—including distinct adipose compartments—obtained from C57BL/6 wildtype mice in order to verify adipose tissue as a major site of Metrnl expression also in the mouse organism. Metrnl mRNA levels (Figure 7) were observed to be highest in adipose tissue and in testicles whereas expression was low in brain, liver, and untrained skeletal muscle. Among different adipose tissue compartments, expression levels were highest in intra-abdominal adipose tissue, followed by subcutaneous and peri-renal adipose tissue. In brown adipose tissue, expression was low yet detectable.

### 3.7. Metrnl Gene Expression Is Transiently Downregulated during 3T3-L1 Adipocyte Differentiation

**Figure 8:**
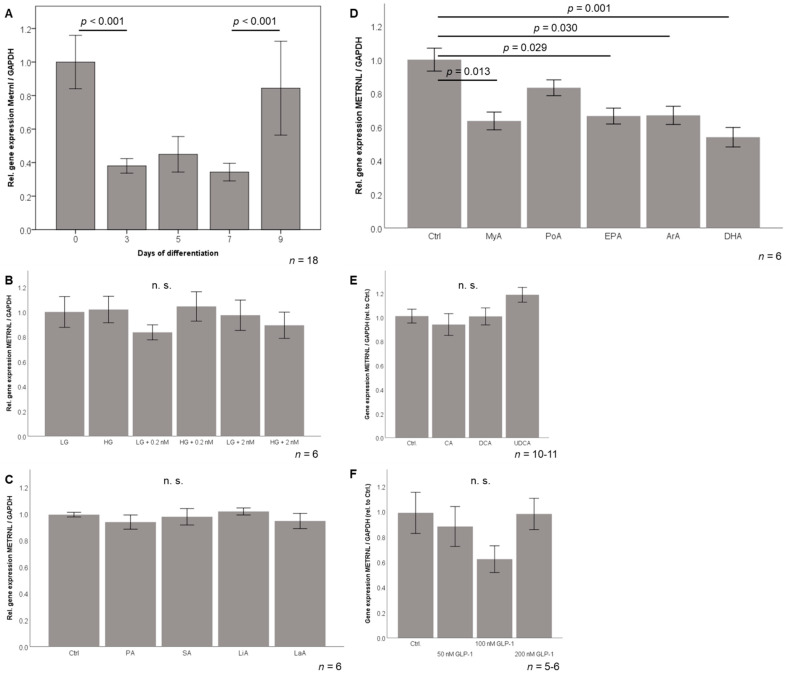
METRNL gene expression during adipocyte differentiation (**A**) and in response to metabolic stimuli (**B**–**F**). GAPDH, glyceraldehyde-3-phosphate dehydrogenase; GLP-1, glucagon-like peptide-1; HG, high glucose concentration (25 mM); LG, low glucose concentration (5.56 mM); Metrnl, Meteorin-like protein; nM, nanomolar insulin concentration. Fatty acids: ArA, arachidonic acid (10 µM); DHA, docosahexaenoic acid (10 µM); EPA, eicosapentaenoic acid (10 µM); LaA, lauric acid (100 µM); LiA, linoleic acid (10 µM); MyA, myristic acid (100 µM); OA, oleic acid (10 µM); PA, Palmitic acid (100 µM); PoA, palmitoleic acid (10 µM); SA, stearic acid (100 µM). Bile acids: CA, cholic acid (100 nM); DCA, deoxycholic acid (10 nM); UDCA, ursodeoxycholic acid (50 nM).

Metrnl mRNA levels were determined by RT-PCR during the hormonally induced differentiation of 3T3L-1 pre-adipocytes into mature adipocytes. Metrnl mRNA expression was high in undifferentiated, fibroblast-like pre-adipocytes and in mature adipocytes at day 9 of differentiation. Metrnl expression was significantly decreased (by ~60% of initial expression) during the process of differentiation (Figure 8A).

### 3.8. Effects of Metabolic Stimuli on Metrnl Expression in Adipocytes

Since the hormonal differentiation protocol mentioned above contained high glucose and insulin concentrations, mature 3T3-L1 adipocytes were investigated under serum-free conditions and exposed to low/high glucose and to low/high insulin concentrations (Figure 8B). Interestingly, Metrnl expression was not sensitive to these metabolic stimuli suggesting that the observed downregulation of Metrnl during differentiation is not mediated by these stimuli.

Since adipocytes represent the most important lipid-storing cell-type, a broad spectrum of dietary/nutritional fatty acids was investigated for possible effects on Metrnl expression (Figure 8C,D). Importantly, most of the tested nutritional fatty acids—saturated (C12, C16, C18), mono-unsaturated (C16, C18), and poly-unsaturated (C18)—did not modulate Metrnl expression. In contrast, saturated (C14) myristic acid (*p* = 0.013) as well as the poly-unsaturated, omega-6 (C20:4) arachidonic acid (*p* = 0.029), omega-3 (C20:5) eicosapentaenoic acid (*p* = 0.030), and omega-3 (C22:6) docosahexaenoic acid (*p* = 0.001) significantly downregulated Metrnl gene expression in mature adipocytes (Figure 8D) suggesting a possible impact of inflammatory changes after surgery. Since the very early decrease of Metrnl immediately after surgery occurred at the same time point when systemic bile acids and incretins have been reported to increase [38,39], we aimed to test in vitro whether bile acid species and the incretin hormone GLP-1 are able to inhibit Metrnl expression. As shown in Figure 8E, the three bile acid subspecies cholic acid (primary bile acid), deoxycholic acid (secondary cholic acid), and ursodeoxycholic acid (therapeutically used, synthetic, tertiary cholic acid) had no effect on adipocytic Metrnl expression when applied in pre-tested, non-toxic doses in adipocytes. Similarly, the incretin hormone GLP-1 (Figure 8F) and also GIP (data not shown) did not modulate Metrnl expression.

## 4. Discussion

The present study provides longitudinal data on serum Metrnl concentrations in two large and well-characterized cohorts of morbidly obese patients. To the best of our knowledge, this is the first study that describes and compares the dynamics of circulating Metrnl concentrations during massive weight loss induced by either low calorie diet or bariatric surgery. Baseline circulating Metrnl levels prior to the start of weight-reducing interventions were equal in male and female patients and were not correlated with BMI, fat mass, or percentage extent of weight loss. However, the expression of Metrnl mRNA in subcutaneous adipose tissue was positively correlated with BMI and the expression of Metrnl mRNA in visceral adipose tissue was positively correlated with the waist-hip-ratio. The observation that only tissue mRNA expression but not the circulating protein correlates with measures of obesity might be explained by the hypothesis that other cellular sources than white adipocytes contribute to the systemic protein concentrations. Screening of gene expression in various mouse tissues verified that Metrnl is abundantly expressed in several adipose compartments (intra-abdominal > subcutaneous > peri-renal > brown adipose tissue). Of note, the differences in Metrnl mRNA levels between intra-abdominal/visceral and subcutaneous compartments are in contrast to our findings in human adipose tissue from the obesity cohort. Furthermore, we also detected Metrnl expression in brain, liver, muscle, and testicles. In contrast to muscle cells post-exercise [13], resting muscle cells only express low levels of Metrnl that ranges below adipose tissue compartments. Overall, we conclude from the present data that Metrnl can be regarded as a classical adipokine and also a neurotrophic protein in mice, with adipose tissue and brain as major sites of gene expression.

Base-line Metrnl levels were found to be significantly decreased in type 2 diabetic patients and were shown to correlate negatively with HbA1c levels in the cohort of LCD patients. Since bariatric patients had a considerably higher average BMI than LCD participants (53.36 vs. 43.48 kg/m^2^), this finding might exclusively exist in the LCD cohort. Within this cohort, a trend of a negative correlation between circulating Metrnl levels and HbA_1c_ was observed for T2D patients but not for normoglycemic individuals. Considering the very low proportion of T2D patients in the LCD study cohort, the physiological impact of this observation remains a matter of speculation.

Interestingly, Metrnl exhibited a significant and positive correlation with serum HDL concentrations and a negative correlation with serum triglycerides. Since nutritional fatty acids did not inhibit adipocytic Metrnl expression, the interpretation of these correlations remains of speculative nature and should be further investigated. Of note, the observed correlation of baseline Metrnl with HDL and triglyceride levels did not persist during weight loss.

A panel of classical and immune-regulatory adipokines and growth factors was measured in both cohorts of patients at baseline. Interestingly, we found a highly significant and positive correlation of Metrnl concentrations with the pro-inflammatory adipokines leptin and resistin in both cohorts. However, Metrnl did not correlate with systemic CRP levels. Thus, Metrnl serum levels might be associated with adipose tissue-related, local inflammation and but not with systemic inflammation.

Both in the LCD and in the bariatric subgroup, patients experienced a significant increase of Metrnl serum levels 3 months after the begin of diet or bariatric surgery, respectively. Thus, circulating Metrnl concentrations appear to be positively associated with the onset of weight loss and this increase occurs independently of the mode of the weight loss-inducing intervention. Since Metrnl concentrations return to the baseline levels after 6 and 12 months, there seem to exist yet unknown compensatory or counter-regulating mechanisms.

Most interestingly and specific to bariatric surgery, we documented a significant and transient decrease of Metrnl immediately after surgery within 3–5 days. Since weight loss does not occur that early, we suggest that other mechanisms are responsible for this effect. As shown, neither glucose nor insulin in different concentrations alone or together were able to modulate adipocyte Metrnl expression, at least in adipocytes. Moreover, a broad panel of tested dietary fatty acids failed to modulate Metrnl expression, whereas mainly pro- and anti-inflammatory, polyunsaturated, omega-3 and omega-6 fatty acids inhibited Metrnl expression in adipocytes. Thus, inflammatory processes post-surgery might be of interest. Moreover, well-known short-term mediators after bariatric surgery such as increasing systemic bile acids and dysregulated incretins such as GLP-1 might have caused the early decrease of Metrnl. Since these substances have been shown to be regulated postprandially and to modulate adipocytic functions [40,41] and adipokine release [42,43], we stimulated adipocytes in vitro with these mediators. However, neither bile acid species nor the incretin hormone GLP-1 were able to downregulate Metrnl expression, at least in adipocytes.

While the present study provides novel data concerning metabolic effects on adipocyte Metrnl expression under defined and serum-free conditions in vitro, a potential regulatory impact of serum components influencing cellular proliferation processes remains an open question. This issue should be addressed by future approaches elaborating on the present data, e.g., in cell culture assays applying serum-conditioned media.

## 5. Conclusions

Metrnl is an adipokine that is differentially regulated during murine adipocyte differentiation. In humans, Metrnl expression is on equal levels in visceral and subcutaneous adipose tissue and circulating Metrnl protein concentrations are not correlated with adipose tissue gene expression suggesting additional sources of Metrnl secretion that contribute to the systemic and circulating protein quantities. Massive weight loss induced by bariatric surgery or low calorie diet in two large and well-described cohorts of patients transiently upregulates circulating Metrnl concentrations after 3 months with levels returning to baseline after 6 and 12 months. The immediate and short-term decrease of systemic Metrnl concentrations within 3–5 days upon bariatric surgery cannot be explained by weight loss and we could exclude effects of bile acids and incretins. Thus, this effect seems to be mediated by other yet unknown short-term mediators such as inflammatory changes after surgery. Whereas insulin, glucose, and nutritional fatty acids are not able to modulate Metrnl expression, omega-3 and omega-6 fatty acids inhibit Metrnl expression in adipocytes and should be further investigated.

## Figures and Tables

**Table 1:** Anthropometric parameters and baseline Metrnl serum levels in LCD (A) and bariatric surgery patients (B). BMI, body mass index; Metrnl, Meteorin-like protein; SD, standard deviation.

**(A)** **Low Calorie Diet** ***n* = 131**	
Females	88 (67.2%)
Males	43 (32.8%)
Age [years] (±SD)	42.1 ± 12.0
BMI [kg/m^2^] (±SD)	43.5 ± 6.7
Serum Metrnl [pg/mL] (±SD)	1117 ± 378
**(B)** **Bariatric Surgery** ***n* = 181**	
Females	143 (79.0%)
Males	38 (21.0%)
Age [years] (±SD)	39.8 ± 11.1
BMI [kg/m^2^] (±SD)	53.4 ± 6.8
Serum Metrnl [pg/mL] (±SD)	1143 ± 383

**Table 2:** Correlation analysis of baseline Metrnl serum levels with anthropometric and biochemical parameters in LCD (A) and bariatric surgery patients (B). ANP, atrial natriuretic peptide; BMI, body mass index; CAMP, Cathelicidin anti-microbial peptide; CRP, C-reactive protein; CTRP-3, C1q/TNF-related protein-3; FGF, fibroblast growth factor; HbA_1c_, glycohemoglobin; HDL, high-density lipoprotein; LDL, low-density lipoprotein; Metrnl, Meteorin-like protein.

**(A)** **Low Calorie Diet** ***n* = 131**		
**Correlation of serum Metrnl with:**	**rho**	***p***
**Metabolism/Inflammation**		
BMI	+0.049	0.579
Body fat (%)	+0.130	0.145
Glucose	−0.078	0.379
Insulin	−0.113	0.130
HbA_1c_	−0.269	0.002
Total cholesterol	−0.038	0.664
LDL cholesterol	−0.077	0.380
HDL cholesterol	+0.191	0.029
Triglycerides	−0.131	0.137
CRP	−0.051	0.563
**Classical adipokines**		
Adiponectin	+0.135	0.124
Leptin	+0.269	0.002
Resistin	+0.187	0.032
**Novel immune-regulatory adipokines**		
Progranulin	+0.180	0.048
CTRP-3	−0.017	0.845
CAMP	−0.115	0.315
**Fibroblast growth factors**		
FGF19	+0.101	0.259
FGF21	+0.066	0.462
**Natriuretic peptides**		
NT-proANP	−0.048	0.585
**(B)** **Bariatric Surgery** ***n* = 181**		
**Correlation of Serum Metrnl with:**	rho	*p*
**Metabolism/Inflammation**		
BMI	−0.005	0.950
Body fat (%)	+0.045	0.579
Glucose	−0.077	0.308
Insulin	+0.023	0.785
HbA_1c_	−0.100	0.208
Total cholesterol	−0.111	0.158
LDL cholesterol	−0.110	0.162
HDL cholesterol (*n* = 163)	+0.180	0.022
Triglycerides (*n* = 163)	−0.164	0.037
CRP	−0.035	0.643
**Classical adipokines**		
Adiponectin	−0.019	0.804
Leptin	+0.222	0.003
Resistin	+0.316	<0.001
**Novel immune-regulatory adipokines**		
Progranulin	+0.083	0.308
CTRP-3	−0.122	0.105
CAMP	+0.003	0.966
**Fibroblast growth factors**		
FGF19	+0.101	0.180
FGF21	+0.137	0.070
**Natriuretic peptides**		
NT-proANP	+0.095	0.202

**Table 3:** Correlation analysis of baseline Metrnl serum levels with anthropometric and biochemical parameters in LCD (A) and bariatric surgery patients (B). BMI, body mass index; CRP, C-reactive protein; HbA_1c_, glycohemoglobin; HDL, high-density lipoprotein; LDL, low-density lipoprotein; Metrnl, Meteorin-like protein.

**(A) Low Calorie Diet** **(*n* = 80)**	**V3**		**V6**		**V12**	
Correlated Parameters	rho	*p*	rho	*p*	rho	*p*
Body fat (%)	−0.191	0.096	−0.005	0.968	−0.170	0.141
BMI	−0.109	0.340	+0.090	0.427	−0.227	0.043
Glucose					−0.145	0.207
HbA_1c_	+0.006	0.957	−0.218	0.055	−0.149	0.191
Total cholesterol					−0.106	0.351
LDL cholesterol					−0.056	0.623
HDL cholesterol					+0.002	0.987
Triglycerides					−0.142	0.214
CRP	−0.056	0.624	+0.002	0.983	−0.062	0.585
**(B) Bariatric Surgery** **(*n* = 82)**	**V3**		**V6**		**V12**	
**Correlated Parameters**	**rho**	***p***	**rho**	***p***	**rho**	***p***
Body fat (%)	−0.096	0.416	−0.070	0.557	−0.161	0.171
BMI	−0.009	0.936	−0.045	0.691	−0.074	0.509
Glucose					+0.232	0.040
HbA_1c_	−0.004	0.972	−0.010	0.929	+0.107	0.343
Total cholesterol					−0.008	0.945
LDL cholesterol					+0.031	0.782
HDL cholesterol					+0.046	0.687
Triglycerides					+0.096	0.395
CRP	−0.006	0.960	+0.190	0.089	+0.254	0.023

## Data Availability

The data presented in this study are available on request from the corresponding author.
